# Short-Time Ocular Ischemia Induces Vascular Endothelial Dysfunction and Ganglion Cell Loss in the Pig Retina

**DOI:** 10.3390/ijms20194685

**Published:** 2019-09-21

**Authors:** Jenia Kouchek Zadeh, Andreas Garcia-Bardon, Erik Kristoffer Hartmann, Norbert Pfeiffer, Wael Omran, Marion Ludwig, Andreas Patzak, Ning Xia, Huige Li, Adrian Gericke

**Affiliations:** 1 Department of Ophthalmology, University Medical Center, Johannes Gutenberg University Mainz, 55131 Mainz, Germany; norbert.pfeiffer@unimedizin-mainz.de (N.P.); w.omran90@gmail.com (W.O.); adrian.gericke@unimedizin-mainz.de (A.G.); 2 Department of Anesthesiology, University Medical Center, Johannes Gutenberg University Mainz, 55131 Mainz, Germany; a.garciabardon@uni-mainz.de (A.G.-B.); hartmane@uni-mainz.de (E.K.H.); 3 Institute of Vegetative Physiology, Charité-Universitätsmedizin Berlin, Charitéplatz 1, 10117 Berlin, Germany; marion.ludwig@charite.de (M.L.); andreas.patzak@charite.de (A.P.); 4 Department of Pharmacology, University Medical Center, Johanens Gutenberg University Mainz, 55131 Mainz, Germany; xianing@uni-mainz.de (N.X.); huigeli@uni-mainz.de (H.L.)

**Keywords:** I/R injury, retinal arterioles, endothelial dysfunction, ganglion cell loss

## Abstract

Visual impairment and blindness are often caused by retinal ischemia-reperfusion (I/R) injury. We aimed to characterize a new model of I/R in pigs, in which the intraocular pathways were not manipulated by invasive methods on the ocular system. After 12 min of ischemia followed by 20 h of reperfusion, reactivity of retinal arterioles was measured in vitro by video microscopy. Dihydroethidium (DHE) staining, qPCR, immunohistochemistry, quantification of neurons in the retinal ganglion cell layer, and histological examination was performed. Retinal arterioles of I/R-treated pigs displayed marked attenuation in response to the endothelium-dependent vasodilator, bradykinin, compared to sham-treated pigs. DHE staining intensity and messenger RNA levels for *HIF-1α*, *VEGF-A*, *NOX2*, and *iNOS* were elevated in retinal arterioles following I/R. Immunoreactivity to HIF-1α, VEGF-A, NOX2, and iNOS was enhanced in retinal arteriole endothelium after I/R. Moreover, I/R evoked a substantial decrease in Brn3a-positive retinal ganglion cells and noticeable retinal thickening. In conclusion, the results of the present study demonstrate that short-time ocular ischemia impairs endothelial function and integrity of retinal blood vessels and induces structural changes in the retina. HIF-1α, VEGF-A, iNOS, and NOX2-derived reactive oxygen species appear to be involved in the pathophysiology.

## 1. Introduction

Ischemia-reperfusion (I/R) events represent a major reason for various retinal disorders [1,2]. For example, breakdown of retinal blood flow, as observed in central retinal artery occlusion (CRAO), is known to have a deleterious impact on visual acuity after already a short time period and represents an ophthalmic emergency with an incidence of approximately 1 per 100,000 people [3]. The lack of oxygen supply to the retina results in massive visual impairment and often in additional sequelae, such as retinal and vitreous hemorrhage, retinal neovascularization, or neovascular glaucoma [4]. Arterial fibrinolysis has failed to improve the clinical outcome of CRAO compared to conservative treatment (e.g., aspirin, ocular massage) or was even shown to be harmful [5,6]. These studies suggest that deleterious, yet poorly understood, molecular processes are activated already in the early phase of retinal ischemia. Although duration of ischemia is a major factor determining tissue damage [7,8,9], the experimental methods to induce retinal or ocular ischemia differ substantially and may also have an impact on the experimental outcome. For example, raising intraocular pressure by cannulation of the anterior chamber and administering saline solution leads to complete occlusion of retinal and ciliary vessels and represents a favorable method to investigate overall alterations within the ocular system due to complete ischemia. An advantage of this method is that it does not require much equipment or technical expertise. However, this technique may also induce tissue damage by mechanical disruption of cellular structures and direct triggering of reactive oxygen species (ROS) production, which may hamper the interpretation of results [10,11,12]. Other models are based on the administration of endothelin-1 or photosensitive rose bengal to induce a partial, dose-dependent vasoconstriction of blood vessels [8,13,14]. The minimally invasive method of applying rose bengal allows for the investigation of ischemia-related molecular pathways [15,16]. However, since a wide range of ocular diseases is associated with reperfusion injury due to restoration of blood flow [1,17], rose bengal may not be suitable to investigate I/R injury because vascular occlusion is induced permanently with this method. Although application of endothelin-1 represents a non-invasive method to induce endogenous vasoconstriction of vessels, it affects physiological pathways by binding to ET_A_ and ET_B_ receptors, which may induce direct release of cytokines and ROS [18].

The goal of this study was to test a new method to induce transient ocular ischemia by complete blockade of arterial blood flow to the eye and brain in pigs, which represent a large animal model with similar ocular characteristics as humans [19,20,21,22]. We tested the hypothesis that 12 min of complete ischemia are enough to induce vascular dysfunction and retinal tissue damage. Another objective of this study was to determine potential molecular mechanisms that are activated after 12 min of ischemia and 20 h of reperfusion.

## 2. Results

### 2.1. Effects of I/R on Monitoring Parameters

**Figure 1:**
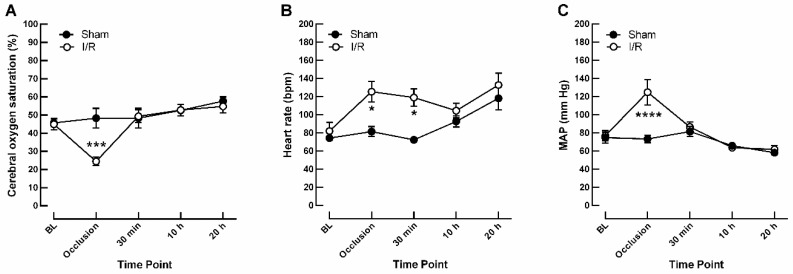
Time courses of cerebral oxygen saturation measured by INVOS™ 5100C (**A**), of heart rate (**B**) and of mean arterial blood pressure (MAP) (**C**) at baseline (BL), during the occlusion time of 12 min (occlusion), 30 min after releasing the occlusion, and after a reperfusion time of 10 and 20 h. Data are expressed as mean ± SE (*n* = 6 per timepoint and group, * *p* < 0.05, *** *p* < 0.001, **** *p* < 0.0001). I/R = ischemia-reperfusion.

At baseline, cerebral oxygen saturation was similar in I/R- and sham-treated pigs (44.9 ± 3.10% and 45.7 ± 2.08%, respectively). During occlusion of vessels supplying the eyes and brain, cerebral oxygen saturation dropped to 24.5 ± 2.33% in the I/R group, while it remained stable at the same time point in the sham group (48.3 ± 5.41%), indicative of ischemia due to reduced blood flow, as shown in Figure 1A. Furthermore, occlusion caused severe tachycardia in the I/R-treated group (125 ± 11.3 bpm versus 81.5 ± 5.52 bpm in I/R versus sham, respectively), as shown in Figure 1B. Mean arterial pressure (MAP) was similar in both groups at baseline but increased in the I/R group when cerebral blood flow was interrupted (125 ± 14.0 mm Hg versus 73.3 ± 4.08 mm Hg in I/R versus sham, respectively), as shown in Figure 1C.

### 2.2. Effects of I/R on Vascular Responses in Retinal Arterioles

**Figure 2:**
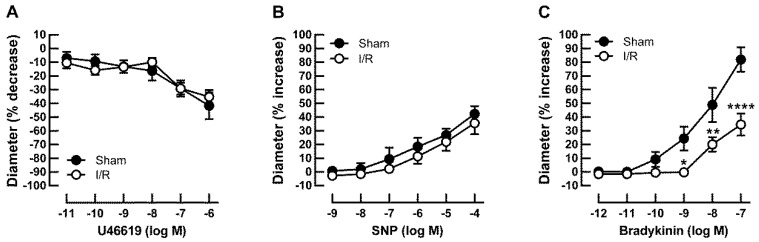
Concentration-dependent responses in retinal arterioles from I/R- and sham-treated pigs to U46619 (**A**), the endothelium-independent vasodilator, SNP (**B**), and to the endothelium-dependent vasodilator, bradykinin (**C**). Data are expressed as mean ± SE (*n* = 6 per concentration and group; * *p* < 0.05, ** *p* < 0.01, **** *p* < 0.0001).

Baseline diameters measured 5 min after cannulation were similar in retinal arterioles from sham- and I/R-treated pigs, as shown in Table 1. Vascular responses in retinal arterioles were measured after development of myogenic tone, which did also not differ between both groups, as shown in Table 1. Vasoconstriction responses to the thromboxane mimetic, U46619, were similar in retinal arterioles from I/R- and sham-treated pigs, as shown in Table 1 and Figure 2A. Likewise, endothelium-independent vasodilatory responses to sodium nitroprusside (SNP) were similar in I/R- and sham-treated pigs, as shown in Table 1 and Figure 2B. In contrast, responses to the endothelium-dependent vasodilator, bradykinin, were impaired in arterioles of the I/R group. Table 1 and Figure 2 show baseline diameters and maximum diameter changes of both groups in response to pharmacological substances used.

### 2.3. Messenger RNA Expression Levels in Isolated Retinal Arterioles

**Figure 3:**
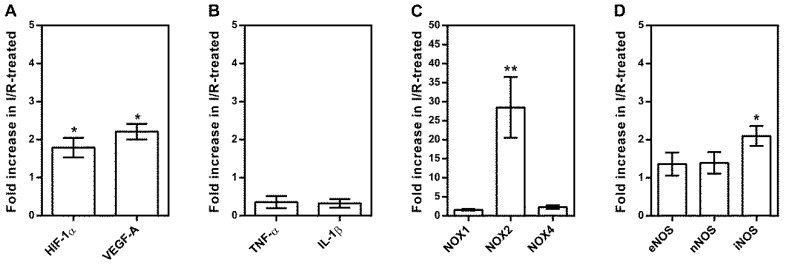
Messenger RNA expression for hypoxic markers (*HIF-1α, VEGF-A*) (**A**), inflammatory cytokines (*TNFα, IL-1ß*) (**B**), prooxidant redox enzymes (*NOX1, NOX2, NOX4*) (**C**), and individual nitric oxide synthase (*NOS*) isoforms (*eNOS, nNOS, iNOS*) (**D**). Data are presented as fold-change in mRNA expression levels in I/R-treated relative to sham-treated pigs. Data are presented as mean ± SE (*n* = 6 per group; * *p* < 0.05, ** *p* < 0.01).

The primer sequences used for PCR analysis are listed in Table 2. In isolated retinal arterioles from I/R-treated pigs, mRNA expression levels were increased for the hypoxic markers, *HIF-1α* and *VEGF-A*, compared to sham-treated pigs *(HIF-1α* ≈ 1.7-fold; * *p* < 0.05 and *VEGF-A* ≈ 2.2-fold, * *p* < 0.05, *n* = 6 per group), as shown in Figure 3A. In contrast, there were no differences in mRNA expression levels for the inflammatory cytokines, *TNF-α* and *IL-1β*, among both groups, as shown in Figure 3B. Furthermore, I/R elicited markedly increased mRNA expression levels for the prooxidant redox enzyme, *NOX2* (≈ 28-fold, ** *p* < 0.01), as shown in Figure 3C. By analyzing mRNA expression levels of all three nitric oxide synthase isoforms (*NOS*), we found that I/R raised expression for inducible NOS (*iNOS*) by ≈ 2.2-fold (* *p* < 0.05), whereas the expression for endothelial and neuronal NOS (*eNOS* and *nNOS*, respectively) did not differ between the two groups, as shown in Figure 3D.

### 2.4. Immunofluorescence

**Figure 4:**
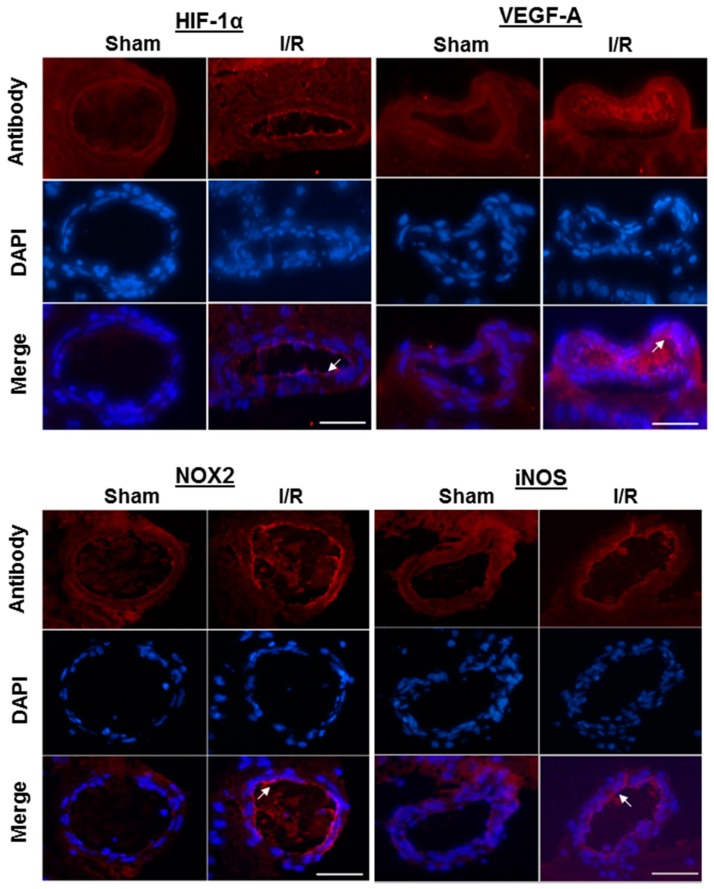
Immunofluorescence micrographs of retinal arteriole cross-sections from sham-treated and I/R-treated pigs stained for HIF-1α, VEGF-A, NOX2, and iNOS. The white arrows point to the endothelial cell layer. Scale bar = 50 μm.

Immunoreactivity to the hypoxic markers, HIF-1α and VEGF-A, to the prooxidant redox enzyme, NOX2, as well as to iNOS was increased in the endothelium of retinal arterioles from I/R-treated pigs, which is in line with the elevated mRNA expression levels found in the arterioles, as shown in Figure 4.

### 2.5. Levels of Reactive Oxygen Species

**Figure 5:**
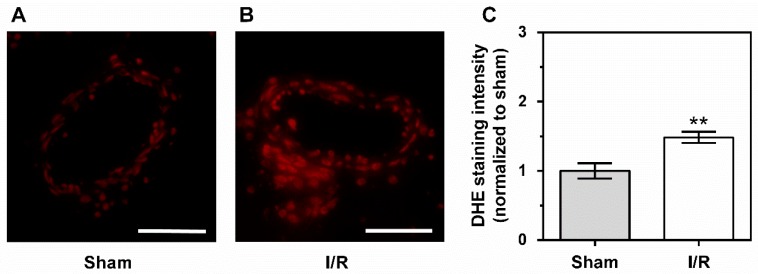
Photomicrographs of dihydroethidium (DHE)-stained retinal arteriole cross-sections from sham- (**A**) and I/R-treated pigs (**B**). DHE staining intensity was markedly increased in I/R-treated pigs (**C**). Data are represented as mean ± SE (*n* = 6 per group; ** *p* < 0.01). Scale bar = 50 μm.

Dihydroethidium (DHE) staining of retinal arteriole cross-sections revealed enhanced fluorescence intensity in the vascular wall of arterioles from I/R-treated pigs, indicative of elevated ROS concentrations compared to sham-treated pigs, as shown in Figure 5.

### 2.6. Cells in the Retinal Ganglion Cell Layer

**Figure 6:**
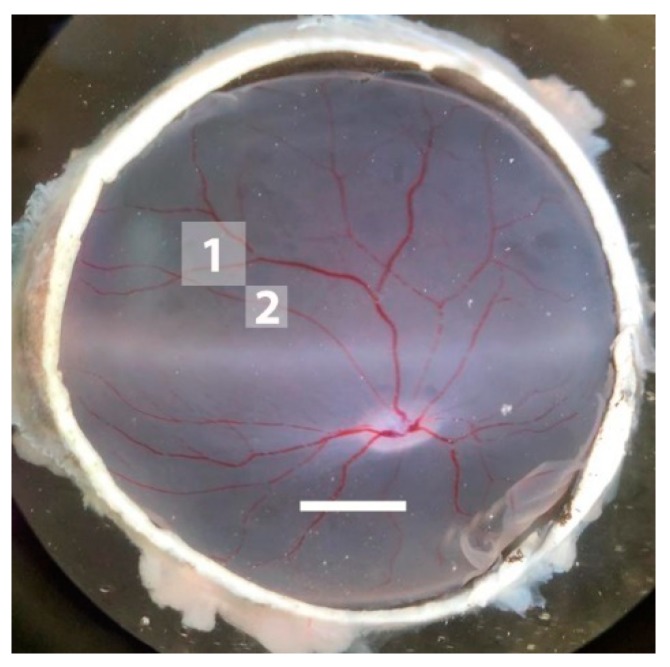
Representative photograph of a pig retina. The marked area labeled with “1” has been used for staining of cells using DAPI and an antibody directed against Brn3a. Just from the area below marked with “2” tissue for histological analysis was taken. Scale bar = 5 mm.

To examine whether cell viability was affected in the retinal ganglion cell (RGC) layer of this I/R model, the density of DAPI-positive cells, which represents the overall cell density in the RGC layer, and the density of Brn3a-positive cells, representing the RGC population, was determined. Figure 6 shows the area selected for cell counting from the midperiphery of the porcine retina.

**Figure 7:**
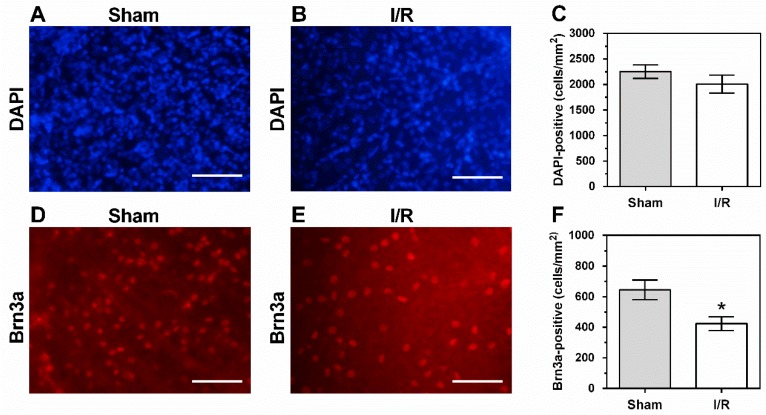
Representative pictures of DAPI-positive cells in sham-treated (**A**) and in I/R-treated pig retinas (**B**). No differences in density of DAPI-positive cells were detected between both groups (**C**). Pictures of Brn3a-stained cells in sham-treated (**D**) and in I/R-treated pigs (**E**). Of note, density of Brn3a-stained cells was markedly reduced in the I/R group (**F**). Data are presented as mean ± SE (*n* = 6 per group; * *p* < 0.05). Scale bar = 100 μm.

The density of DAPI-stained cell nuclei in the RGC layer did not differ between I/R- and sham-treated pigs (2254 ± 130 cells/mm^2^ versus 2009 ± 178 cells/mm^2^), as shown in Figure 7. In contrast, a reduction in density of Brn3a-positive cells by 34.3% was observed in I/R-treated pigs compared to sham-treated pigs (424 ± 45.2 cells/mm^2^ versus 645 ± 64.4 cells/mm^2^), as shown in Figure 7.

### 2.7. Retinal Histology

**Figure 8:**
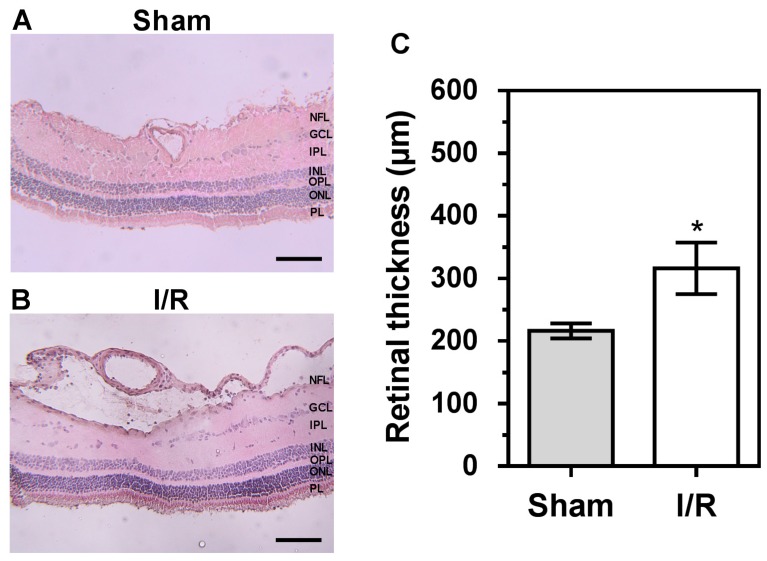
Photomicrographs of retinal cross-sections (H&E staining) from sham-treated (**A**) and I/R- treated pigs (**B**). Quantification of retinal thickness in cryosections revealed increased thickness in I/R-treated pigs (**C**). Data are presented as mean ± SE (*n* = 6 per group; * *p* < 0.05). NFL = nerve fiber layer; GCL = ganglion cell layer; IPL = inner plexiform layer; INL = inner nuclear layer; OPL = outer plexiform layer; ONL = outer nuclear layer; PL = photoreceptor layer. Scale bar = 100 μm.

Compared to retinal tissue from sham-treated pigs, as shown in Figure 8A, retinas of I/R- treated pigs displayed fluid accumulation in the nerve fiber layer, especially localized around the arterioles, as shown in Figure 8B. In addition, derangement of the arterial wall architecture was visible in retinal arterioles from the I/R group, suggesting a disturbed vascular integrity. In addition, the RGC layer appeared disorganized in the I/R-treated group. Retinal thickness was markedly increased following I/R-treatment compared to sham-treatment (318 ± 41 µm versus 216 ± 12 µm, respectively), as shown in Figure 8C.

## 3. Discussion

There are several new findings in this study. First, we present a new porcine model of ocular ischemia by inducing global cerebral ischemia. Remarkably, 12 min of ischemia and 20 h of reperfusion resulted in marked endothelial dysfunction of retinal arterioles. Second, ROS levels and mRNA expression levels for *HIF-1α*, *VEGF-A*, *NOX2,* and *iNOS* were elevated in retinal arterioles and immunoreactivity to HIF-1α, VEGF-A, NOX2, and iNOS was increased in retinal arteriole endothelium. Third, a significant loss of RGCs and morphological changes of the retina were observed following I/R. To the best of our knowledge, this is the first model on pigs investigating the effects of short-time ischemia on retinal vascular function and retinal morphology. The findings suggest that retinal tissue is very susceptible to already short periods of ocular ischemia.

A variety of ischemic models in brain research has already been established, starting from cerebral occlusion time periods of 10 min [23] over 30 min to up to 2 h [24], all of them resulting in neuronal ischemic damage of brain structures. In contrast, the time period resulting in irreversible retinal damage remains controversial due to a variety of experimental designs and methods across different species. Of note, retinal ischemia can be triggered by two vessel occlusion (2VO), also termed bilateral common carotid arteries occlusion (BCCAO), leading to a 50% retinal ischemia, as well as by combined occlusion of vertebral arteries and common carotid arteries, also called four vessel occlusion (4VO), resulting in complete retinal ischemia of 95–100% [25], which is the case in the present study.

In the current study, we observed endothelial dysfunction of retinal arterioles and structural changes in retinal tissue of I/R-treated pigs. Recent studies have been more and more focused on the association between endothelial dysfunction and the severity of vision-threatening diseases like primary open angle glaucoma and diabetic retinopathy due to the important role of an intact endothelium for proper regulation of retinal perfusion [26,27,28]. In the present study, retinal arteriole dilation after I/R was blunted in response to the endothelium-dependent vasodilator, bradykinin, but not to the endothelium-independent vasodilator, SNP, indicative of endothelial dysfunction. Since NOX2 expression and ROS production were increased in retinal arterioles of I/R-treated pigs, oxidative stress appears to be a trigger factor for endothelial dysfunction. As a consequence of ischemic injury and retinal artery occlusion, retinal damage was previously shown to come along with an increased expression of the hypoxic marker, HIF-1α, and its target genes VEGF, NOX2, and iNOS [14,29], which is supported by the present study. Moreover, several studies suggested iNOS to contribute to the pathophysiology of diabetic retinopathy [30,31]. Furthermore, upregulation of iNOS in endothelial cells induced by I/R was associated with apoptosis, cell migration, and endothelial cell dysfunction [32]. To further search for potential sources of ROS in this model, we investigated expression levels of prooxidant NOX enzymes, which were shown to be involved in various retinal diseases [15,33]. Remarkably, mRNA expression for *NOX2* was elevated in retinal arterioles and immunoreactivity to NOX2 was enhanced in the retinal arteriole endothelium of I/R-treated pigs, suggesting this enzyme to be a potential source of ROS and involved in the onset of endothelial dysfunction in our model. Various laboratories, including our own, have shown a link between hypoxia-induced HIF-1α upregulation and enhanced NOX2 expression in other disease models [34,35,36]. Moreover, the retinal edema, which we observed in the vicinity of arterioles from I/R-treated pigs suggests that already 12 min of ocular ischemia are sufficient to induce a noticeable disruption of the inner blood–retinal barrier, which is in line with the upregulated VEGF-A expression. We have previously observed similar changes in a porcine model of acute respiratory distress syndrome [34]. VEGF increases vascular permeability and promotes pathological neovascularization in various ocular diseases, such as age-related macular degeneration and diabetic retinopathy, and its expression is also regulated by HIF-1α during hypoxic conditions [37]. A loss of vascular integrity was also shown by increased leakage of sodium fluorescein tracers and infiltration of monocytes into the ischemic eye at 72 h after ischemic stroke using a 90 min transient middle cerebral artery occlusion (MCAO) model in mice [38]. The authors also observed a delayed inflammatory response by increased mRNA levels for proinflammatory cytokines, such as *TNF-α*, after 90 min of ocular ischemia and 8 h of reperfusion, which is in disagreement with our model, but may be explained by differences in species characteristics and the duration of ischemia.

To assess whether retinal neuron viability was affected after 12 min of ischemia and 20 h of reperfusion, we determined overall cell density in the RGC layer by counting DAPI-positive cells and RGC density by counting Brna3a-positive cells. In the retina, Brn3 transcription factors, including Brn3a, are exclusively expressed in RGCs [39]. Although we did not find marked changes in overall cell density, we observed a decrease in RGC density by ≈34% in I/R-treated pigs. These findings suggest that the subgroup of RGCs may be especially vulnerable to I/R. Since the density of Brna3a-positive cells was only 28.6% and 21.1% of the overall cell density in the RGC layer of sham- and I/R-treated pigs, respectively, selective loss of RGCs in the I/R group may have remained undetected when looking only at the overall cell density. The density of Brn3a-positive cells in the sham group was close to previously reported porcine RGC density data of the same retinal region obtained by retrograde labeling of RGCs with Fluoro-Gold [40], suggesting that the Brn3a antibody bound specifically to RGCs. Moreover, the overall cell density was in line with previously reported cell density data obtained by the Nissl staining method [41].

Bardy et al. demonstrated hemodynamic changes and impairment of cell function in miniature pigs by experimental microembolization for 10 min only, which is supported by the present study [42]. A study by Osborne et al. has shown in a rat model of BCCAO that 24 min of blood flow cessation followed by 6 days of reperfusion enhanced glial fibrillary acidic protein expression in the retina, suggesting that this relatively short period of ischemia already affected retinal tissue homeostasis [43].

In contrast to these findings, a series of studies by Hayreh et al. on primates revealed that CRAO elicited retinal damage not before a duration of 105 min, indicative of a high retinal tolerance to ischemic injury [44,45]. One explanation for the discrepancies between the studies may be the extent and type of occlusion of vessels supplying the retina. In those animal models, where the retinal circulation was selectively blocked, residual oxygen and glucose supply to the retina may be maintained by the choroidal network. Conversely, in the models with complete ocular ischemia, including the present one, the choroidal supply is also blocked, which may be a reason for the shorter ischemic tolerance of retinal tissue. Another explanation may be species differences with regard to retinal vascular supply and ischemic tolerance [46]. Although porcine eyes constitute an established and validated model in vision research due to high morphological resemblance to human eyes, e.g., with respect to the mean area of retina and vascularization, ischemia-induced retinal damage is still poorly understood in pigs [20,21].

In conclusion, complete cerebral ischemia presents a feasible method to induce ocular ischemia without direct manipulation of the eye. A disadvantage of this method is that it requires a high level of technical effort and expertise. A major new finding of this study is that only 12 min of ocular ischemia followed by a reperfusion period of 20 h induced endothelial dysfunction in retinal arterioles, retinal thickening, indicative of edema due to vascular leakage, and RGC loss. Hypoxia-induced changes, such as upregulation of HIF-1α, VEGF-A, NOX2, and iNOS, as well as oxidative stress, appear to be involved in the pathophysiology.

## 4. Materials and Methods

### 4.1. Animals

All experimental protocols were approved by the Animal Care Committee of Rhineland-Palatinate, Germany (date of approval: 11 June 2013), and adhere to the EU Directive 2010/63/EU for animal experiments. Male German Landrace pigs (*Sus scrofa domesticus*, 12–16 weeks, 33–36 kg) were obtained from a local farm and sedated for transport by an intramuscular injection of azaperone and ketamine (4 mg/kg). After arrival at the research facility, anesthesia was induced by intravenous injection of fentanyl (4 μg/kg), propofol (3 mg/kg), and atracurium (1.5 mg/kg) via an ear vein cannula and maintained by continuous infusion of fentanyl (10 μg/kg/h) and propofol (6 mg/kg/h). After endotracheal intubation, volume-controlled ventilation was maintained with the following settings: tidal volume 8 mL/kg; positive end-expiratory pressure 5 cm H_2_O, FiO_2_ = 0.3; inspiration to expiration ratio 1:2; and variable respiration rate to achieve an end-tidal pCO_2_ < 6 kPa. Rectal temperature was continuously monitored, and body temperature was maintained using a heating blanket throughout the experiment. Arterial and venous catheters were placed via ultrasound guidance into the femoral vessels for central venous vascular access and invasive blood pressure monitoring. Hemodynamic parameters, such as arterial pressure and heart rate, were continuously measured. Cerebral oxygen saturation (rSO_2_) was quantified with a self-adherent near infrared spectroscopy probe placed bilaterally on the forehead. The rSO_2_ values were updated and displayed in five-second intervals with the INVOS™ 5100C Cerebral/Somatic Oximeter (Somanetics Corporation, Troy, MI, USA), providing a highly sensitive real-time parameter for changes in cerebral blood flow.

### 4.2. Materials

Components for the Krebs–Henseleit buffer were obtained from Carl Roth GmbH, Karlsuhe, Germany. The vasodilator, bradykinin (Sigma-Aldrich Chemie GmbH, Steinheim, Germany; purity ≥98%), induces endothelium-dependent vasodilation in various blood vessels, including porcine retinal arterioles [47,48]. The endothelium-independent vasodilator, sodium nitroprusside (SNP; Sigma-Aldrich; purity ≥99%) is a donor of nitric oxide (NO) [49]. The vasoconstrictor, U46619 (Cayman Chemical, Ann Arbor, MI, U.S.; purity ≥98%), is a thromboxane A2 (TP) receptor agonist [50]. The stock solution of U46619 was dissolved in dimethyl sulfoxide (DMSO), whereas bradykinin and sodium nitroprusside were dissolved in phosphate buffered saline (PBS).

Antibodies against NOX2 (ab129068, 1:100), VEGF-A (ab9570, 1:100), and iNOS (ab15323, 1:100) were purchased from Abcam, Berlin, Germany. The antibody directed against HIF-1α (NB100-654, 1:100) was obtained from Bio-Techne GmbH, Wiesbaden, Germany. For the NOX2, HIF-1α, and VEGF-A antibodies used in this study, we have previously shown an increased immunoreactivity in retinal arteriole endothelium of hypoxic pigs with acute respiratory distress syndrome [34]. The secondary antibody was coupled with Rhodamine Red-X (111-035-045, 1:200) and was purchased from Dianova GmbH, Hamburg, Germany. For immunostaining of RGCs, a goat polyclonal brain-specific homebox/POU domain protein 3A (Brn3a) antibody purchased from Santa Cruz Biotechnology (sc-31984, Santa Cruz, CA, USA, 1:750) was used. The antibody was directed against an epitope close to the N-terminus of the human Brn3a protein and was shown to be suitable for RGC detection in rats and mice [51,52]. The amino acid sequence of this region is identical in humans, pigs, mice, and rats. In pilot experiments performed in porcine retinal-cross sections, we found positive immunoreactivity only in a portion of cells localized in the RGC layer, suggesting specific binding to RGCs. Donkey anti-goat IgG Alexa Fluor 568 (A11057, Life Technologies, Carlsbad, CA, USA 1:400) was used as secondary antibody.

### 4.3. Surgical Procedure

Cerebral ischemia was induced in six pigs (I/R group). After sternotomy, carotid and right vertebral inflow were occluded by clamping the innominate artery, containing right and left carotid and right subclavian artery, just distal of the aortic arch. The left subclavian artery was clamped equally proximal to occlude inflow via the left vertebral artery. Effective clamping and consecutive cerebral ischemia were confirmed by an INVOS™ 5100C Cerebral/Somatic Oximeter (Medtronic GmbH, Meerbuch, Germany) and by an increase of blood pressure and heart rate, as well as by dilated non-reactive pupils. After 12 min, clamping was released, and blood flow restored. After initial stabilization, pigs were monitored, and normal hemodynamic parameters were maintained for the next 20 h. Sham surgery was also conducted in six pigs. In this group, apart from the occlusion of arteries, the same surgical preparation was conducted as in the I/R group.

### 4.4. Measurement of Vascular Reactivity in Retinal Arterioles

After pigs had been monitored for 20 h following I/R, they were sacrificed by inducing cardiac arrest via application of high doses of propofol (200 mg) and potassium chloride (40 mmol) intravenously. Next, the eyes were enucleated and transferred into ice-cold Krebs–Henseleit buffer of the following ionic composition (in mM): 118.3 NaCl, 4.7 KCl, 2.5 CaCl2, 1.2 MgSO4, 1.2 KH2PO4, 25 NaHCO3, 11 glucose. After opening the eye globe, the retina was carefully isolated as described previously [34]. Retinal arterioles of the first order were then isolated and cleaned from surrounding retinal tissue by Vannas scissors and fine-point tweezers. Vascular measurements were conducted after cannulation of blood vessels onto two micropipettes as described previously [53,54]. Only when the luminal arteriole diameter decreased by at least 30% in response to 100 mM KCl, the vessel was used for experiments. Concentration–response curves were started after development of basal tone, which was achieved after an equilibration time of 45 min. A myogenic tone of 30%–50% of the initial diameter was defined as preconstricted for the following concentration–response curves to vasodilators. If not achieved, the thromboxane mimetic, U46619 (Cayman Chemical, Ann Arbor, MI, U.S.), was titrated into the circulating Krebs–Henseleit buffer to achieve proper preconstriction.

### 4.5. Measurement of Reactive Oxygen Species

Retinal arterioles together with surrounding retinal tissue were isolated immediately after enucleation, embedded in Tissue Tek OCT compound (Sakura Finetek Europe, Alphen aan den Rijn, Netherlands), frozen in liquid nitrogen, and stored at −80 °C until use. For staining, cryosections of 10 µM thickness were placed on Superfrost Plus slides (Thermo Fisher Scientific, Menzel-Gläser, Braunschweig, Germany) and 1 mL of 5 µM dihydroethidium (DHE, Thermo Fisher Scientific, Waltham, MA, U.S.) solution was dropped onto each slide. Then, all slides were placed in a light-protected and humidified chamber and incubated at 37 °C for 30 min. Oxidized DHE sections were analyzed as described previously [34,55].

### 4.6. Quantitative PCR

Directly after enucleation, vessels were isolated in cold Krebs–Henseleit buffer using fine-point tweezers and microscissors, washed in cold phosphate buffered solution (PBS, Thermo Fisher Scientific, Braunschweig, Germany), transferred into a 1.5 mL tube, and snap-frozen. Homogenization of tissue was performed in lysis buffer (1.0% NP40, 0.5% sodium deoxycholate, 0.1% SDS, 10 mmol/l NaF, 80 mmol/l TRIS, pH 7.5). Quantitative PCR was performed according to the manufacturer’s protocol by using a light cycler (LC480, Roche Diagnostics, Mannheim, Germany) and a StepOnePlus device (Applied Biosystems, Foster City, CA, USA). SYBR Green (Thermo Fisher Scientific) was utilized for fluorescent detection of DNA generated during PCR. Relative mRNA levels were quantified using comparative threshold (CT) normalized to the β-actin gene. Primer sequences are presented in Table 2.

### 4.7. Immunohistochemistry

Retinal tissue containing first-order retinal arterioles was excised for immunohistochemical evaluation and embedded in Tissue Tek OCT compound (Sakura Finetek Europe). After freezing in liquid nitrogen, the tissue was stored at −80 °C until use. Frozen sections of 10 μm thickness were cut and fixed in 4% paraformaldehyde (pH 7.4) solution for 20 min. Next, slides were rinsed with PBS and incubated at room temperature with blocking solution containing 0.1% Triton-X-100 and 0.1% bovine serum albumin for 30 min. Next, primary antibodies directed against HIF-1α, VEGF-A, NOX2, and iNOS were diluted in blocking solution and incubated for 2 h at room temperature. Thereafter, each slide was washed in PBS three times for 5 min and incubated for 1 h at room temperature with a secondary Rhodamine Red-X-coupled antibody (Dianova GmbH). For negative controls, the primary antibody was omitted.

For immunohistochemical visualization of RGCs, retinas were carefully separated from the pigment epithelium by injection of Krebs buffer. In each retina, the optic disc was used as a reference point. To minimize localization-dependent variations in RGC density, we have chosen a retinal piece of 3 × 3 mm localized in the nasal superior midperiphery of the retina. The lower temporal corner of this area was localized 7 mm nasally and 7 mm superior to the optic disc center, as shown in Figure 6. We have chosen this area because the retinal midperiphery represents the major part of the porcine retina containing ≈70% of the RGCs and has a relatively homogeneous RGC density [40]. A pair of compasses was used to localize and measure the area, which was harvested for cell staining. After careful excision of the retinal piece and fixation with 4% paraformaldehyde for 30 min, the tissue was washed in PBS + 0.5% Triton-X-100 twice for 10 min and then frozen for 15 min at −80 ° C. Following this procedure, the tissue was thawed at room temperature and then washed twice with PBS and 0.5% Triton-X-100. A primary antibody for Brn3a, an established tool for RGC staining [51,52], was diluted in blocking buffer containing PBS + 2% Triton-X-100 + 2% fetal calf serum (FCS). After incubation overnight at 4 °C, residual antibody was removed by washing the tissue three times for 10 min with PBS + 0. 5% Triton-X-100. Then, retinal tissue was incubated with the secondary antibody for 2 h at room temperature. Subsequently, the tissue was washed three times for 10 min with PBS and mounted with 4’,6-diamidino-2-phenylindole (DAPI)-containing medium (VECTASHIELD^®^ Mounting Medium with DAPI, H-1200, BIOZOL Diagnostica Vertrieb GmbH, Eching, Germany) and cover-slipped. Next, in each stained piece of retina, 10 areas, each representing 0.138 mm^2^, were photographed by fluorescence microscopy. Brn3a- and DAPI-positive cells were counted in all 10 areas manually and semi-automatically using a macro [56] installed in ImageJ cell counting software (version 1.52a) [57]. The counting procedure included the following steps: convert to 8-bit, subtract background, auto threshold, run nucleus counter (smallest 800, largest 7000) as described elsewhere [58]. The mean cell number was then determined for the 10 counted areas per piece of tissue, and the cell density per mm^2^ calculated.

### 4.8. Retinal Histology

Retinal cryosections of 10 µM thickness, each containing a cross-section of a first-order retinal arteriole taken from an area 2 mm superior to the visual streak, as shown in Figure 6, were fixed with 4% paraformaldehyde (Histofix, Roth, Karlsruhe, Germany) for 20 min at room temperature. Next, tissue sections were washed with purified water twice for 5 min, immersed in hematoxylin for 3 min, and washed another time with purified water for 5 min. Sections were placed into 95% ethanol for 1 min, followed by 1 min of staining in eosin solution. Subsequently, tissue dehydration was done by ascending ethanol series (70%, 96%, and 100%) and washing in xylene (3 × 5 min). Subsequently, the glass slides were mounted with Eukitt quick-hardening mounting medium (Sigma-Aldrich, Steinheim, Germany) and visualized by transmitted light microscopy (Nikon, Yurakucho, Tokyo, Japan). Retinal thickness was measured at five standardized positions, and the average was calculated for each retinal cross section.

### 4.9. Statistical Methods

Time courses of tissue oxygenation, heart rate, and mean arterial pressure, as well as concentration–response curves, were compared by two-way analysis of variance (ANOVA) for repeated measurements and the Sidak’s multiple comparisons test. A two-sided unpaired t-test was used to compare ROS levels, mRNA expression levels (ΔCT values), cell density, and retinal thickness. Data are presented as mean ± SE, and n represents the number of pigs per group. The significance level was set at 0.05.

## 5. Conclusions

In conclusion, we developed a new porcine model, which induces endothelial dysfunction of retinal arterioles by ocular and global brain ischemia and leads to retinal pathological changes, such as edema and loss of RGCs after only 12 min of ischemia and 20 h of reperfusion. Moreover, our data suggest that the HIF-1α-VEGF-A-NOX2 pathway plays a crucial role in inducing retinal vascular endothelial dysfunction in the early phase of ocular ischemia.

## Figures and Tables

**Table 1:** Initial diameter (baseline) 5 min after cannulation; basal arteriolar tone after 45 min of equilibration (myogenic tone) in % from baseline. Maximum vascular diameter changes of retinal arterioles to the thromboxane mimetic, U46619, to sodium nitroprusside (SNP) and to bradykinin in % from baseline (myogenic tone). Data are presented as means ± SE.

		Diameter Changes [%]			
	Baseline [µm]	Myogenic Tone [%]	U46619 at 10^−6^ M[%]	SNP at 10^−4^ M [%]	Bradykinin at 10^−7^ M [%]
**Sham**	85.5 ± 20.6	−46.1 ± 5.79	−41.6 ± 9.89	42.3 ± 5.62	82.0 ± 8.85
**I/R**	91.8 ± 24.7	−42.7 ± 4.18	−35.2 ± 5.11	35.6 ± 8.06	34.5 ± 8.02

**Table 2:** Primer sequences for mRNA expression studies.

Gene Name	Primer Sequence
*NOX1*	F: TCAGTTTTATTTCTGGCTGCTTGG R: CTTTCTCAGGGTGCGCCTAC
*NOX2*	F: CACTTCACGCCACGATTCAC R: TTGACTCGGGCGTTCACAC
*NOX4*	F: GTCCCAGTGTGTCTGCGTTAG R: TCTCGAAATCGTTCTGTCCAGTC
*eNOS*	F: CTACAGGACCCAAGATGGGC R: TGAAGCAGGGTACAGGGTCT
*nNOS*	F: ATTTTCGGAGTCACCCTGCG R: AGCTGAAAACCTCATCTGTGTCT
*iNOS*	F: ACTATTTCTTCCAGCTTAAGAGCC R: CTCGTAGGGAAATACAGCACCA
*TNF-α*	F: TTCTGCCTACTGCACTTCGAG R: TGAGACGATGATCTGAGTCCTT
*IL-1* *β*	F: ATAGTACCTGAACCCGCCAAG R: GTGCAGCACTTCATCTCTTTGG
*HIF-1α*	F: CGTGCGACCATGAGGAAATG R: GTGAAGTACTTTCCATGTTGCAG
*VEGF-A*	F: ATAGAGCGAGGCAAGAAAATCCC R: ACACGTCTGCGGATCTTGTA
*β* *-actin*	F: TGGACTACCTCCTGTCTGCT R:CCTAGGGGTGGGTTTCTGTG

## Abbreviations

BL	Baseline
Brn3a	Brain specific homebox/POU domain protein 3A
CRAO	Central retinal artery occlusion
DAPI	4’,6-diamidino-2-phenylindole
DHE	Dihydroethidium
DMSO	Dimethyl sulfoxide
eNOS	Endothelial nitric oxide synthase
FCS	Fetal calf serum
GCL	Ganglion cell layer
HIF-1α	Hypoxia-inducible factor-1α
HR	Heartrate
iNOS	Inducible nitric oxide synthase
INL	Inner nuclear layer
IL	Interleukin
IPL	Inner plexiform layer
I/R	Ischemia-reperfusion
MAP	Mean arterial blood pressure
NFL	Nerve fiber layer
nNOS	Neuronal nitric oxide synthase
NOX	Nicotinamide adenine dinucleotide phosphate oxidase
ONL	Outer nuclear la
OPL	Outer plexiform layer
PBS	Phosphate-buffered saline
RGC	Retinal ganglion cell
ROS	Reactive oxygen species
SNP	Sodium nitroprusside
TNF- α	Tumor necrosis factor alpha
U46619	9,11-dideoxy-9α,11α-methanoepoxy prostaglandin F2α
VEGF-A	Vascular endothelial growth factor
